# Hippocampal-Sparing Radiation Therapy in Primary Sinonasal and Cutaneous Tumors of the Head and Neck

**DOI:** 10.1016/j.adro.2024.101588

**Published:** 2024-08-24

**Authors:** Jacob Hall, Michael Dance, Benjamin Huang, Ethan Steele, Lorie Nguyen, Michael Repka, Xuguang Chen, Colette Shen

**Affiliations:** a Department of Radiation Oncology, University of North Carolina, Chapel Hill, North Carolina; b Department of Radiology, University of North Carolina, Chapel Hill, North Carolina; c University of North Carolina, Chapel Hill, North Carolina

## Abstract

**Purpose:**

Patients with primary sinonasal and cutaneous head and neck (H&N) malignancies often receive meaningful radiation dose to their hippocampi, but this not a classic avoidance structure in radiation planning. We aimed to characterize the feasibility and tradeoffs of hippocampal-sparing radiation therapy (HSRT) for patients with primary sinonasal and cutaneous H&N malignancies.

**Methods and Materials:**

We retrospectively selected patients who were treated definitively for primary sinonasal or cutaneous malignancies of the H&N at an academic medical center. All received (chemo)radiation alone or adjuvantly and substantial radiation dose to 1 or both hippocampi. We created new HSRT plans for each patient with intensity modulated radiation therapy using the original target and organ-at-risk (OAR) volumes. Hippocampi were contoured based on Radiation Therapy Oncology Group guidelines and reviewed by a neuroradiologist. Absolute and relative differences in radiation dose to the hippocampi, planning target volumes (PTVs), and OARs were recorded and compared.

**Results:**

There were 18 sinonasal and 12 cutaneous H&N primary tumors (30 patients in total). Median prescription dose was 6600 cGy (range, 5000-7440 cGy), and 14 of the 30 patients received 120 cGy/fraction twice daily, 13 of the 30 patients received 200 cGy/fraction once daily, whereas others received 180-275 cGy/fraction once daily. The relative decrease in ipsilateral hippocampal D_max_ and D100% using HSRT was 44% (median, 2009 cGy from 3586 cGy) and 65% (median 434 cGy from 1257 cGy), respectively. There were no statistically significant or clinically meaningful differences in PTV V100%, PTV D1%, or radiation dose to other OARs between HSRT and non-HSRT plans.

**Conclusions:**

HSRT is feasible and results in meaningful dose reduction to the hippocampi without reducing PTV coverage or increasing dose to other OARs. We suggest target hippocampal constraints of D_max_ < 1600 cGy and D100% < 500 cGy when feasible (without compromising PTV coverage or impacting other critical OARs). The clinical significance of HSRT in patients with primary H&N tumors should be investigated prospectively.

## Introduction

Radiation therapy represents a cornerstone of therapy for patients with primary head and neck malignancies, whether definitively for those that are locally advanced or in locations that are not amenable to surgical resection (eg, the nasopharynx or certain sinonasal cavity tumors) or adjuvantly for patients with higher-risk pathologic features. Intensity modulated radiation therapy (IMRT) has allowed for the dosimetric sparing of many normal tissues in the head and neck compared with 3-dimensional conformal radiation therapy, reducing the level of normal tissue toxicity.^1^, ^2^, ^3^ Despite these advances, patients still often experience bothersome toxicity related to radiation therapy. Among these side effects, especially for patients whose treatments affect greater volumes of the brain, is neurocognitive decline including worse memory, depression, and worse language ability.^4^, ^5^, ^6^, ^7^, ^8^ Several studies have investigated cognitive decline in patients with head and neck cancer treated with definitive radiation therapy, 2 of which report results using IMRT and suggest a correlation between cognitive decline and radiation therapy dose to the temporal lobes.^4^^,^^5^

The hippocampus is a structure in the medial temporal lobe that is known to be important for learning and memory and has demonstrated regenerative capacity, even throughout adulthood.^9^^,^^10^ Radiation sensitivity of precursor cells involved in neurogenesis has been shown in preclinical models.^11^, ^12^, ^13^ Prospective studies have demonstrated an improvement in various measures of cognitive function when limiting radiation dose to the hippocampi for primary or metastatic brain tumors.^14^^,^^15^ However, the hippocampi are not intentionally avoided with radiation during trials studying the benefits of IMRT compared with 3-dimensional conformal radiation therapy in head and neck cancer^1^^,^^2^ and are not routinely avoided as part of standard head and neck cancer radiation therapy.

The hippocampus receives a clinically significant dose of radiation during a typical radiation therapy course for certain head and neck primary tumors. This has been shown in patients with primary tumors located in the nasopharynx.^16^, ^17^, ^18^ Several retrospective studies have demonstrated the feasibility of avoiding the hippocampi during IMRT planning without compromising target volume coverage. These studies included patients with primary tumors of the nasopharynx, oral cavity, and oropharynx (locally advanced).^17^, ^18^, ^19^, ^20^, ^21^ However, studies demonstrating feasibility for primary skin malignancies located in the head and neck and sinonasal tumors are lacking, to our knowledge. Sharma et al^22^ studied late neurocognitive functioning in patients with sinonasal cancer treated with radiation and found a correlation between hippocampal dose and memory and executive function. This suggests efforts to limit radiation dose to the hippocampus may improve late neurocognitive function in these patients.

Our retrospective cohort includes patients with primary sinonasal and cutaneous tumors of the head and neck. Our objective was to demonstrate the feasibility of reducing radiation dose to the hippocampi without compromising target coverage or significantly increasing dose to other traditionally avoided organs-at-risk (OAR) in this cohort.

## Methods and Materials

### Patient selection

This retrospective case series was approved by the institutional review board (The University of North Carolina IRB) and performed at a single institution. We retrospectively selected patients with primary sinonasal and cutaneous tumors of the head and neck treated with definitive or postoperative radiation therapy based on diagnosis codes. Each patient received photon radiation at our institution from 2017-2021. We aimed to include 30 total patients representing a variety of tumors fitting these criteria. Each patient included received D1% of >16 Gy and/or D100% of >5 Gy to 1 or both hippocampi, which was felt to be a significant dose considering these constraints have been or are being prospectively studied. For patients with primary skin malignancies, the tumor was generally located at a similar axial level as the hippocampi, which caused the hippocampi to receive significant radiation dose. Generally, cutaneous tumors for patients fitting these criteria were located on the forehead, ears, and temporal regions. Patients with primary skin malignancies treated with electrons were excluded given the limited dose range of electrons and subsequent limited dose to the hippocampus.

### Radiation planning—previous treatment and hippocampal avoidance

For radiation treatment planning, targets and OARs were contoured and IMRT plans were created using RayStation (RaySearch Laboratories). Hippocampi were not contoured or avoided as part of original treatment planning. We retrospectively contoured the bilateral hippocampi after identifying patients and original radiation plans for the study. Contours for previous target volumes and OARs were unchanged. For patients with magnetic resonance imaging (MRI) available, T1-weighted diagnostic brain MRI scans were rigidly fused to the computed tomography (CT) simulation scan at the time of initial treatment planning. Hippocampi were contoured based on the Radiation Therapy Oncology Group (RTOG) hippocampal atlas, and no planning OAR volume was used. In cases when MRI was not available, the hippocampi were contoured using CT simulation scan only. IMRT-based hippocampal-sparing radiation therapy (HSRT) plans were created with target coverage being the top priority while limiting radiation dose to the hippocampi without substantially increasing dose to other OARs. Target constraints for the hippocampi were D100% of ≤5 Gy and D_max_ (D0.1 mL) of ≤16 Gy.^15^^,^^23^

### Analysis

We compared radiation doses with the ipsilateral or contralateral hippocampi between the initial plan and HSRT plan using D_max_ and D100% (defined as the minimum dose delivered to 100% of the contoured hippocampal volume). We used D1% to represent D_max_. All contoured hippocampal volumes were sufficiently small such that 1% of the contoured volume was <0.03 mL, which is commonly used to define D_max_. In the study by Brown et al^15^ in which they measured cognitive outcomes following hippocampal avoidance whole brain radiation therapy, D0.03 mL was used to define D_max_. The ipsilateral hippocampus was defined as the hippocampus receiving the highest radiation dose between the 2. We also compared PTV coverage and dose with other OARs that are standardly measured at our institution. These included the standard-risk and high-risk (HR) PTV (HR PTV) V100%, HR PTV D1%, parotid mean dose, lacrimal mean dose, lens D_max_, optic nerve D_max_, optic chiasm D_max_, cochlea mean dose, brainstem D_max_, whole brain mean dose, and whole brain D_max_. V100% was defined as the volume of the target volume receiving 100% of the prescription radiation dose. For bilateral OARs, the dosimetric variable was averaged for the left and right structures. This averaged value was used to compare OAR doses between HSRT and non-HSRT. A paired samples *t* test was used to compare hippocampal D_mean_, D_max_, and D100%, dosimetric variables for other OARs, and HR PTV V100% and PTV D1% between the original and HSRT plans.

## Results

We included 30 patients in our analysis. The median age at the time of radiation was 65 years (range, 27-88 years). Eighteen of these patients had primary sinonasal tumors, and 12 had primary cutaneous tumors of the head and neck. Twelve of 18 primary sinonasal tumors were histologies with applicable T staging, and the distribution is in Table 1. Sixteen patients (53%) had MRI available for contouring, while the simulation CT was used for hippocampal contouring in the remaining 14. For those with MRI available, the median bilateral hippocampal volume was 1.6 mL (range, 0.8-2.6 mL). For patients for whom CT-based contouring was used, the median bilateral hippocampal volume was 1.9 mL (range, 0.7-3.4 mL). The median HR PTV for all patients was 164.5 mL (range, 51-1321 mL). Twenty-five of 30 patients were also treated with standard-risk volumes. Median standard-risk PTV was 628 mL (range, 262-1817 mL). The most common prescription radiation dose and fractionation was 60 Gy in 30 fractions. Additional patient characteristics are displayed in Table 1.

**Table 1:** Patient and tumor characteristics

	No. of Patients
Sinonasal substructure	
Maxilla	9
Nasal cavity	8
Frontal/ethmoid sinuses (Tx77)	1
Skin	12
Sinonasal T stage	
T4	7
T3	3
T2	2
T1	0
Prescription dose (cGy)/No. of fractions	
6960/58	5
6600/33	2
7440/62	4
6480/54	4
6000/30	8
5000/25	2
7000/35	1
6000/50	1
5500/20	1
5400/30	1
5000/20	1
No. of patients treated twice a day	14/30
No. of patients with MRI available for contouring	16/30

*Abbreviations:* MRI = magnetic resonance imaging.

**Figure 1:**
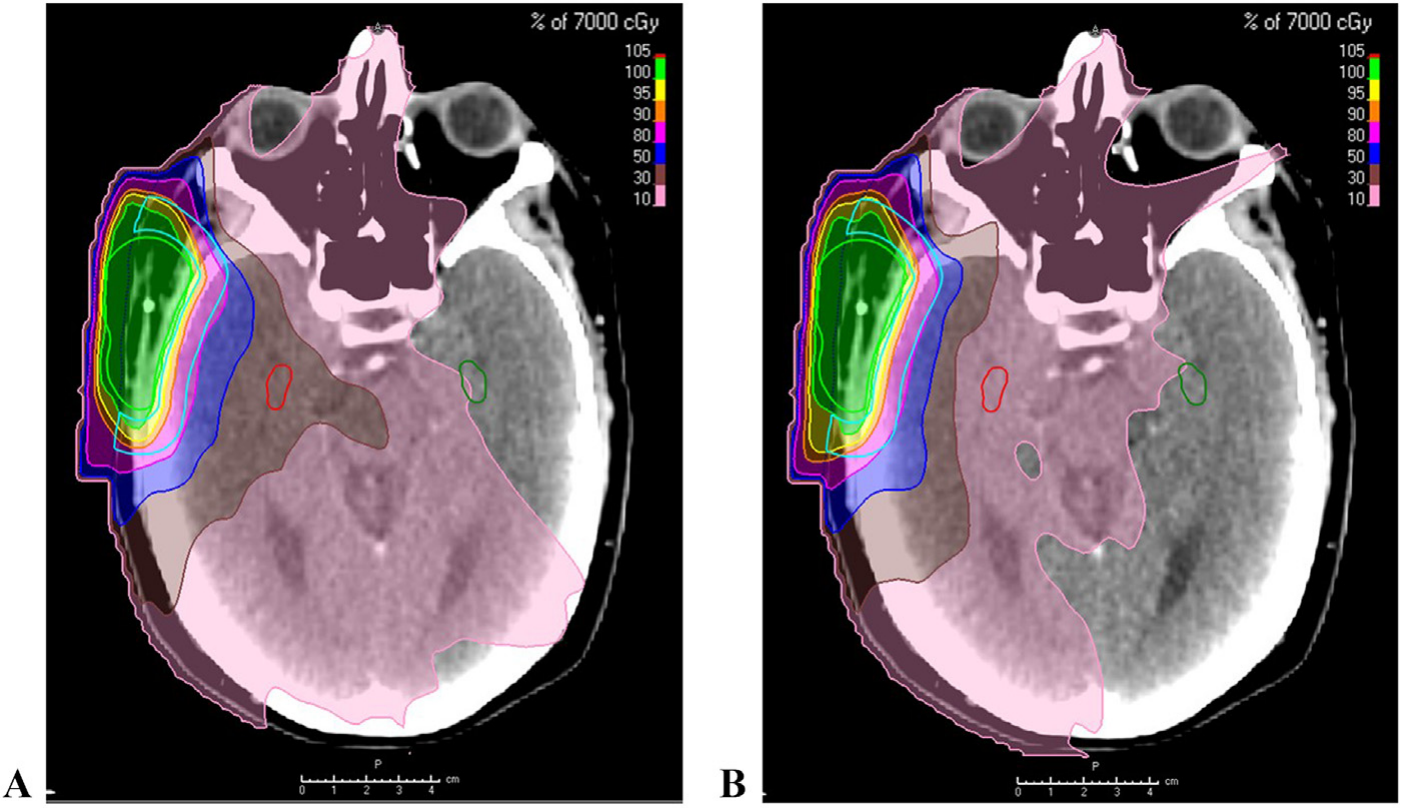
A patient with a cutaneous basal cell carcinoma of the postauricular region: (A) radiation therapy plan without HSRT; (B) radiation therapy plan with HSRT. *Abbreviations:* HSRT = hippocampal-sparing radiation therapy.

**Figure 2:**
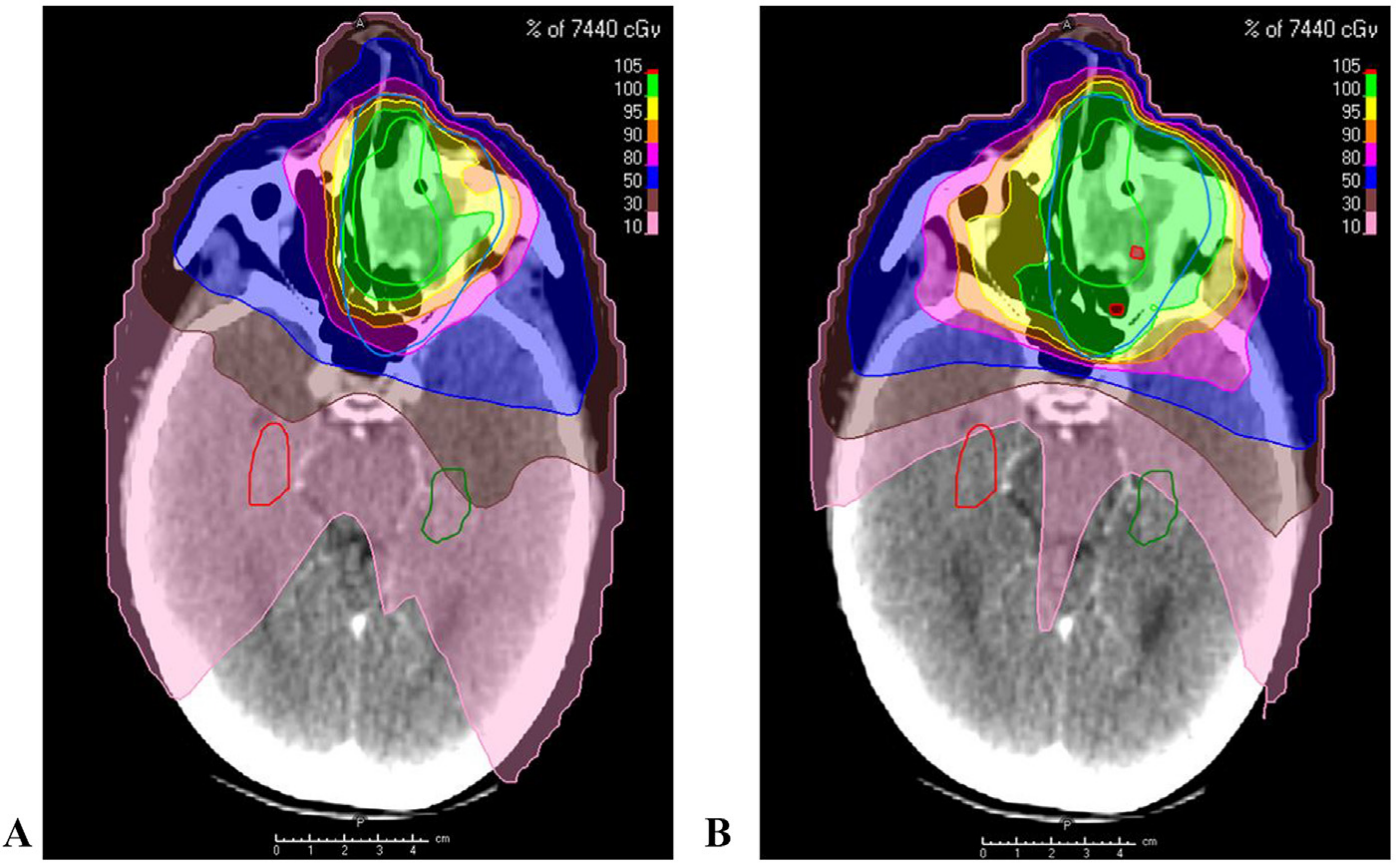
A patient with a primary sinonasal malignancy: (A) radiation therapy plan without HSRT b) radiation therapy plan with HSRT *Abbreviations:* HSRT = hippocampal-sparing radiation therapy.

There was a relative decrease in ipsilateral hippocampus median D_mean_, D_max_, and D100% of 56%, 44%, and 65% (*P* <.001), respectively, using HSRT compared with the original plan. The relative decrease of contralateral hippocampus median D_mean_, D_max_, and D100% was 50%, 45%, and 50% (*P* <.001), respectively, using HSRT compared with the original plan. Absolute median values are displayed in Table 2. Thirteen of the 30 (43%) and 24 of the 30 (80%) patients achieved D_max_ of ≤16 Gy with HSRT for ipsilateral and contralateral hippocampi, respectively. Nineteen of the 30 (63%) patients and 28 of the 30 (93%) patients achieved D100% of ≤5 Gy with HSRT for ipsilateral and contralateral hippocampi, respectively. Figure 1, Figure 2 illustrate the improvement in hippocampal dose distribution using HSRT for a patient with postauricular basal cell carcinoma treated with postoperative radiation therapy (Fig. 1) and a patient with cT4N0M0 squamous cell carcinoma of the left frontal sinus treated with concurrent chemoradiation following induction chemotherapy (Fig. 2).

**Table 2:** Differences in radiation dose variables of the hippocampi and PTV with and without HSRT

	Dose without HSRT (cGy), median (range)	Dose with HSRT (cGy), median (range)	Relative difference (%; *P*)
Ipsilateral hippocampus D_mean_	1923 (529-4240)	845 (317-2724)	−56 (<.001)
Ipsilateral hippocampus D_max_	3586 (769-6018)	2009 (456-4673)	−44 (<.001)
Ipsilateral hippocampus D100%	1257 (184-2659)	434 (150-2426)	−65 (<.001)
Contralateral hippocampus D_max_	2046 (358-4321)	1125 (258-3043)	−45 (<.001)
Contralateral hippocampus D100%	708 (104-2242)	353 (91-1808)	−50 (<.001)
PTV HR V100% (%)	95.0	95.0	0.0 (.352)
PTV SR V100% (%)	98.4	98.2	−0.2 (.327)
PTV D1%	6829 (5235-7915)	6740 (5205-7880)	−1.3 (.160)

*Abbreviations:* HR = high risk; HSRT = hippocampal-sparing radiation therapy; PTV = planning target volume; SR = standard risk.

There were no significant differences in dosimetric variables describing PTV radiation dose between HSRT and non-HSRT plans. For all OAR dosimetric variables measured, only the median lacrimal glands mean dose and median whole brain D_max_ increased (by 2.4% and 1.2%, respectively) following HSRT when compared with the original plan. Median dosimetric variables for all other OARs decreased on average by 4.6% to 10%. Absolute values with ranges and relative changes are displayed in Table 3.

**Table 3:** Differences in radiation dose variables of OARs

	Dose without HSRT (cGy), median (range)	Dose with HSRT (cGy), median (range)	Relative difference (%; *P*)
Parotid glands mean dose	2130 (84-6257)	2004 (79-6252)	−5.9 (.001)
Lacrimal glands mean dose	948 (202-5062)	971 (188-5108)	2.4 (.069)
Lenses mean dose	2029 (341-5777)	1831 (323-5650)	−9.7 (.007)
Optic nerves D_max_	4077 (564-6467)	3790 (416-6511)	−7.0 (.057)
Optic chiasm D_max_	4685 (611-6524)	4354 (492-5824)	−7.1 (.007)
Cochlea mean dose	1942 (108-6258)	1792 (108-6261)	−7.7 (.001)
Brainstem D_max_	3946 (783-5962)	3765 (538-5900)	−4.6 (.001)
Whole brain mean dose	1435 (374-3408)	1285 (299-3347)	−10 (.001)
Whole brain D_max_	6577 (5128-7492)	6654 (5094-7441)	1.2 (.523)

*Abbreviations:* HSRT = hippocampal-sparing radiation therapy; OAR = organ-at-risk.

## Discussion

The hippocampi often receive meaningful radiation dose in patients receiving definitive or adjuvant (chemo)radiation therapy for certain tumors of the head and neck, particularly primary nasopharyngeal, sinonasal, and cutaneous tumors of the head and neck. Previous series have demonstrated meaningful hippocampal radiation dose in primary nasopharyngeal tumors and have shown the feasibility of HSRT.^17^^,^^18^^,^^21^^,^^24^ We demonstrated similar findings in our study: hippocampi receive meaningful radiation dose when treating locally advanced sinonasal and cutaneous tumors of the head and neck. Second, and more importantly, it is feasible to limit this radiation dose below constraints used in prospective protocols without comprising target coverage or significantly increasing dose to other traditionally measured OARs.

### Hippocampal sparing—potential benefits

Preclinical models have demonstrated the sensitivity of cells involved in hippocampal function to radiation and subsequent cognitive dysfunction, even with low doses.^23^^,^^25^ Monje et al^11^ found there was inhibition of precursor proliferation and neurogenesis in a mouse model following whole brain radiation therapy (WBRT). One study showed apoptosis of proliferating stem cells responsible for neurogenesis in the hippocampus following a single 10-Gy dose in rats, and another showed human neural stem cells are sensitive to even lower doses (<10 Gy).^12^^,^^13^ Although the clinical impact of low-dose radiation to these cells has not been demonstrated, the sensitivity of cells related to hippocampal function to low-dose radiation has. Moving to more clinically applicable studies, there are few prospective clinical trials measuring the cognitive benefit of HSRT at various radiation doses. Gondi et al^14^ showed worse delayed verbal recall in patients who received an equivalent dose in 2-Gy fractions of >7.3 Gy to 40% of the bilateral hippocampi in a prospective study. The randomized study by Brown et al^15^ also showed improved cognitive function in patients receiving hippocampal avoidance WBRT plus memantine versus those receiving standard WBRT plus memantine. Hippocampal dose constraints in the study by Brown et al^15^ were D100% of ≤9Gy and D_max_ (0.03 mL) of ≤16Gy for the bilateral hippocampi. In a recent prospective protocol investigating hippocampal avoidance in low-grade gliomas, investigators selected D100% of ≤5 cobalt gray equivalents.^23^ Our hippocampal dose constraints were selected based on dose constraints from the abovementioned prospective protocols and clinical evidence.^15^^,^^23^ We felt limiting the radiation dose to a stricter constraint than D100% of ≤9 Gy may be beneficial in a population being treated with curative intent, especially if it is possible without negatively impacting other radiation plan metrics.

### Hippocampal sparing—tradeoffs

Maintaining a constant integral dose, sparing the hippocampi will lead to increased dose elsewhere in the same axial plane. Dunlop et al^21^ created hippocampal-sparing radiation plans for 10 patients with primary nasopharyngeal cancer and demonstrated this phenomenon. Dose difference maps from this study showed radiation dose increases along the midline, bilateral anterolateral temporal lobes, and in the maxillary sinuses at the axial level of the hippocampi. The increase in dose was generally ≤6 Gy, which is unlikely to be clinically significant.^21^ However, this has not been confirmed prospectively and may be more of a concern in cases where doses to the temporal lobes exceed 60 Gy and increase the risk of radionecrosis. We know limiting hippocampal radiation dose improves cognitive function to some extent, but limiting radiation dose in other regions of the temporal lobe may also affect cognitive function. Further determining which regions of the brain are less affected by radiation and have a smaller effect on cognition is integral to optimizing cognitive function following radiation, rather than simply avoiding the hippocampus.

Our study showed statistically significant reductions in hippocampal median D_mean_, D_max_, and D100% for both ipsilateral and contralateral sides without compromising target coverage or increasing other OAR radiation dose. There were statistically significant reductions in other OAR radiation doses using HSRT (aside from the hippocampi), but we do not intend to portray HSRT as a method for doing so. These changes were small and clinically insignificant. HR PTV V100% was 95% for both the original and HSRT plans. We expect this and acknowledge this is a result of prespecified IMRT goals in planning. We feel that HSRT for head and neck primary tumors should routinely be considered even without prospective evidence proving HSRT improves cognitive outcomes in this population, given the very low risk, minimal effort, and potential benefit demonstrated in other patient populations of doing so. The hippocampi are rarely an at-risk region of cancer spread in this population. This recommendation assumes target coverage is maintained and radiation dose to other OARs is not significantly different, including confirming clinically acceptable dose to the anterior temporal lobes.

### Limitations

We acknowledge that the dose constraints used in our study were the same for every patient, regardless of prescription dose and number of fractions. We felt this allowed for additional simplicity because the goal of this study was to demonstrate feasibility rather than to measure cognitive function. Should the benefits of HSRT in patients with head and neck tumors be studied prospectively, we recommend the dose constraints account for the dose and number of fractions being used in the study. In addition, the D_max_ of ≤16 Gy constraint used in this study was based on a radiation dose and fractionation schedule different from most head and neck cancer treatments (total 30 Gy over 10 fractions) but was satisfied for many patients in our study. Should this be adjusted for typical head and neck plans, which often use 30 fractions when conventionally fractionated, the dose constraints would be even less difficult to meet. Another consideration when adjusting dose constraints from a 10-fraction plan to longer, 30+-fraction daily plans or 50+-fraction twice daily plans is that the linear-quadratic model used to calculate equivalent dose in 2-Gy fractions does not account for time.

An additional limitation is that a portion of the patients in this study did not have brain MRI available for hippocampal contouring, requiring us to use the simulation CT. The hippocampal contours were typically larger when using the CT given the anatomic uncertainty, which would theoretically make it more difficult to meet hippocampal constraints. However, there may be some situations where anatomic inaccuracies caused by hippocampal contouring on CT could lead to a misleading, more favorable position of the hippocampal contour relative to high radiation dose. In this study, hippocampal contours (including those contoured on the simulation CT without MRI) were reviewed and edited by a board-certified neuroradiologist (BH) at our institution (example CT-based hippocampal contours in Fig. E1).

## Conclusions

Limiting radiation dose to the hippocampi can reduce the risk of neurocognitive decline. Definitive and adjuvant (chemo)radiation for primary sinonasal and skin cancers in the head and neck can deliver substantial incidental dose to the hippocampi. We demonstrated feasibility in limiting radiation dose to the hippocampi below previously validated constraints used for WBRT without comprising target coverage or increasing meaningful dose to other OARs. This simple and resource-efficient technique should be used when the hippocampi may receive substantial incidental radiation dose in effort to minimize neurocognitive toxicity. Better quantification of neurocognitive toxicity because of hippocampal radiation for head and neck primary tumors should be studied prospectively to allow physicians and patients to understand better the benefits of HSRT.

## Disclosures

Colette Shen reports research support from AstraZeneca (not related to this work) and is a consultant for Nanobiotix and GT Medical Technologies (unrelated to this work). All authors except for Lorie Nguyen are employed by the University of North Carolina.

## Appendix Supplementary materials

Supplemental Figure 1 Image, image 1

## Footnotes

Supplementary material associated with this article can be found in the online version at doi:10.1016/j.adro.2024.101588.
