# Supplementary figures and images for: Hippocampal-Sparing Radiation Therapy in Primary Sinonasal and Cutaneous Tumors of the Head and Neck
Source: Adv Radiat Oncol. 2024 Aug 24;9(10):101588. doi: 10.1016/j.adro.2024.101588 (PMC11387223; doi:10.1016/j.adro.2024.101588)

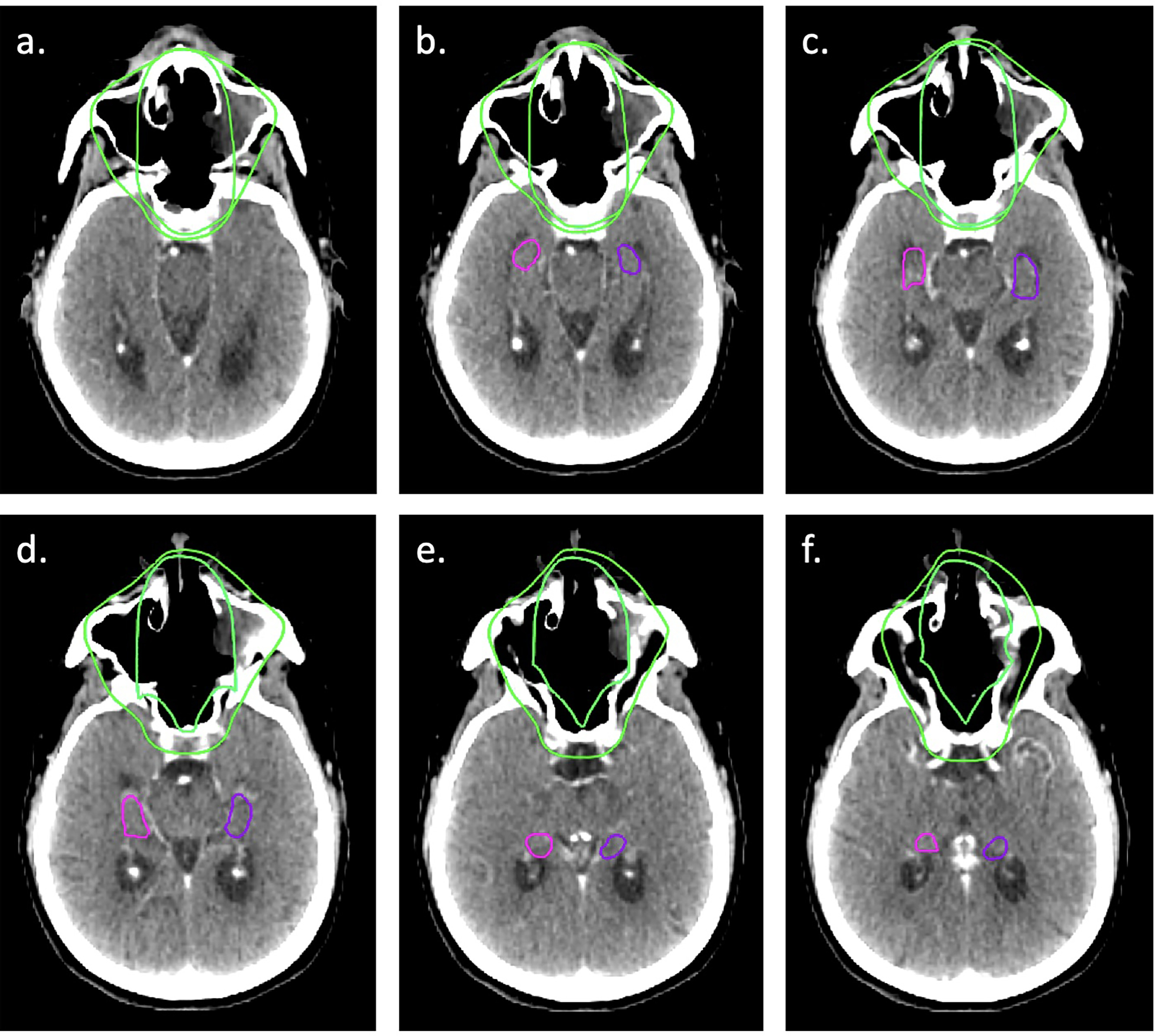

Supplement: Supplemental Figure 1 [file mmc1.jpg]
